# Enhanced Production of Recombinant Protein by Fusion Expression with Ssp DnaB Mini-Intein in the Baculovirus Expression System

**DOI:** 10.3390/v10100523

**Published:** 2018-09-25

**Authors:** Won Seok Gwak, Jae Bang Choi, Beom Ku Han, Sung Min Bae, Soo Dong Woo

**Affiliations:** 1 Department of Agricultural Biology, College of Agriculture, Life & Environment Science, Chungbuk National University, Cheongju 28644, Korea; Wonseok1031@cbnu.ac.kr; 2 Optipharm Inc., Osong 28158, Korea; jbc@optipharm.co.kr (J.B.C.); bkhan@optipharm.co.kr (B.K.H.); 3 Diagnostic Development 2 Team, ImmuneMed Inc., Chuncheon 24232, Korea

**Keywords:** baculovirus, Ssp DnaB mini-intein, fusion partner, purification, enhanced production

## Abstract

The baculovirus expression system (BES) is considered to be a very powerful tool for the expression of numerous difficult to express vertebrate proteins. Ssp DnaB mini-intein is a useful fusion partner for the production of recombinant proteins because it can be self-cleaved by controlling the pH and temperature, without additional treatment. To evaluate the utility of Ssp DnaB mini-intein in the BES, recombinant viruses were generated to express the enhanced green fluorescent protein, the VP2 protein of porcine parvovirus, and the E2 protein of classical swine fever virus fused to a mini-intein. As expected, intracellular self-cleavage of the mini-intein occurred during virus infection, but the cleavage initiation time varied depending on the target protein. Significantly enhanced protein production was observed for all of the target proteins that were fused to the mini-intein. This increase was enough to overcome the decrease in the fusion protein due to intracellular self-cleavage. The mini-intein in all of the recombinant fusion proteins was successfully cleaved by controlling the pH and temperature. These results suggest that the Ssp DnaB mini-intein is a useful fusion partner in the BES for easy purification and enhanced production of target proteins.

## 1. Introduction

The baculovirus expression system (BES) is widely used for the production of vertebrate proteins or vaccines in insect cells or larvae. The BES is less expensive than mammalian cell expression systems, has a convenient protein production method, and has a short production time [1]. The most useful feature of the BES is that it leads to the expression of correctly folded and post-translationally modified proteins at a similar level to mammalian expression systems [2,3]. The BES uses a *polyhedrin* promoter with strong activity. However, the production efficiency of a foreign protein using this *polyhedrin* promoter is not as high as that of native polyhedrin [4]. Therefore, various attempts have been made to increase the production efficiency of foreign proteins, such as the addition of transcriptional enhancers or fusion with a carrier protein as a “fusion partner”. The fusion partner is frequently highly expressed in host cells, which not only enhances the expression level of the fusion protein, but also favors the purification of the fusion protein [5]. However, these methods have not been applied to all proteins; thus, it is necessary to remove the fusion partner to prevent structural and functional changes to the target protein [6,7]. Therefore, enzymes such as a TEVp protease [8], TAGZyme [9], or enterokinase [10] should be used, although they have the disadvantage of requiring an additional process for the removal of the protease [6]. Additionally, these enzymes are expensive, difficult to store, and may even degrade the target protein, so special care is required.

An intein with 429 amino acids was discovered in the DnaB gene, encoding a DNA helicase of *Synechocystis* sp. PCC6803 [11]. The Ssp DnaB mini-intein is a splicing proficient minimal intein consisting of N-terminal 106 residues and C-terminal 48 residues by deletion of the central 275 amino acids [12]. This mini-intein has been well used in commercial plasmids such as the IMPACT system (New England Bio labs, Ipswich, MA, USA). Peptide bond hydrolysis between the C-terminal region of the Ssp dnaB mini-intein and target protein is triggered by shifting the pH from 6.0 to 7.5 at room temperature [13]. Therefore, the target protein can easily be separated from the fusion partner or an affinity tag without an additional protease treatment [14]. Thus, this system is an efficacious tool for protein engineering, such as protein expression and purification, because of its self-cleavage capability [11,15,16].

Several previous reports have shown that the final yield of the target protein may be low because the Ssp DnaB mini-intein fusion protein undergoes in vivo self-cleavage [17]. Self-cleavage may occur because the temperature and pH of the cell culture are similar to the self-cleavage conditions of the Ssp DnaB mini-intein. The conditions for insect cell culture are very similar to the self-cleavage conditions of the Ssp DnaB mini-intein. However, the Ssp DnaB mini-intein has not been applied to baculovirus expression systems. Thus, in this study, we applied the Ssp dnaB mini-intein to the baculovirus expression system and reported the optimal conditions for its use and the additional effects of the Ssp dnaB mini-intein.

## 2. Materials and Methods

### 2.1. Cells and Viruses

*Bombyx mori* 5 (Bm5) cells were cultured at 27 °C and pH 6.4 in TC-100 insect medium (WelGENE, Gyeongsan, GB, Korea) that was supplemented with 10% fetal bovine serum [18]. The wild-type (K1) and recombinant *B. mori* nucleopolyhedroviruses (BmNPVs) used in this study were propagated in Bm5 cells. The classical swine fever virus (CSFV LOM strain, GenBank accession number EU789580) and porcine parvovirus (PPV VRI-1 strain, GenBank accession number AY390557) provided from Optipharm Inc. were used in this study. Routine cell culture maintenance and virus production procedures were performed according to the published procedures [19].

### 2.2. Construction of Transfer Vector

**Figure 1:**
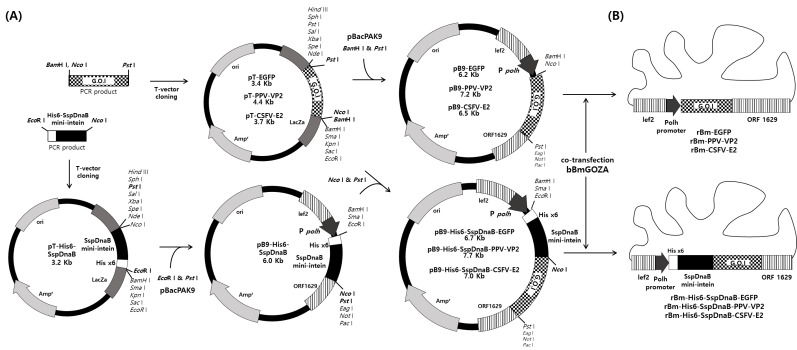
(**A**) Construction of the transfer vectors (**B**) and a schematic representation of recombinant viruses. PCR amplification of each of the DNA fragments that were cloned into the T-vector; the PCR products were sequentially cloned into the transfer vector pBacPAK9. Finally, each constructed transfer vector was co-transfected into Bm5 cells with bBmGOZA to generate each recombinant BmNPV.

The Ssp DnaB mini-intein gene was amplified from the pTWIN1 plasmid (New England Bio labs, Ipswich, MA, USA) with the primers His6-SspDnaB-F and SspDnaB-R (Table 1). The PCR products were directly cloned into the pMD20-T-vector (TaKaRa, Kusatsu, Shiga, Japan) and digested with *Eco*R I and *Pst* I and were then subsequently cloned into the corresponding restriction sites of the pBacPak9 vector (Clontech, Mountain View, CA, USA) to construct pB9-His6-SspDnaB. The enhanced green fluorescent protein (EGFP) gene was amplified from a pEGFP-N1 plasmid (Clontech, Mountain View, CA, USA) with the primers EGFP-F and EGFP-R (Table 1). The PPV *VP2* gene was amplified from PPV with the primers PPV-VP2-F and PPV-VP2-R (Table 1). The CSFV *E2* gene (CSFV-E2-ΔTMR) without transmembrane region (TMR) was amplified from CSFV with the primers CSFV-E2-F and CSFV-E2-R (Table 1). The PCR products were directly cloned into the T-vector and digested with *Nco* I and *Pst* I and were subsequently cloned into the corresponding restriction sites of the pB9-His6-SspDnaB vector to construct pB9-His6-SspDnaB-EGFP, pB9-His6-SspDnaB-PPV-VP2 and pB9-His6-SspDnaB-CSFV-E2 (Figure 1A). To generate control recombinant baculoviruses, the cloned PCR products in the T-vector were digested with *Bam*H I and *Pst* I and subsequently cloned into the corresponding restriction sites of the pBacPak9 vector to generate pB9-EGFP, pB9-PPV-VP2 and pB9-CSFV-E2 (Figure 1).

### 2.3. Generation of Recombinant Virus

Recombinant BmNPVs rBm-His6-SspDnaB-EGFP, rBm-His6-SspDnaB-PPV-VP2, rBm-His6-SspDnaB-CSFV-E2, rBm-EGFP, rBm-PPV-VP2, and rBm-CSFV-E2, expressing each target gene under the control of the *polyhedrin* promoter, were generated by co-transfection of each transfer vector, pB9-His6-SspDnaB-EGFP, pB9-His6-SspDnaB-PPV-VP2, pB9-His6-SspDnaB-CSFV-E2, pB9-EGFP, pB9-PPV-VP2, and pB9-CSFV-E2, and a defective viral genome, bBmGOZA DNA (Figure 1B) [20]. Transfection was performed using the Cellfectin II™ (Invitrogen, Carlsbad, CA, USA) reagent according to the manufacturer’s instructions, and the recombinant viruses were purified and propagated in Bm5 cells as previously described [20].

### 2.4. Measurement of Fluorescence

To measure the fluorescence intensity, rBm-His6-SspDnaB-EGFP was infected into Bm5 cells. The infection was carried out with 1 × 10^6^ cells per well in 6-well plates that were infected with a multiplicity of infection (MOI) of 5 PFU/cell. Infected cells were collected at 24 h intervals from 24 to 120 hours post-infection (h.p.i) and washed with ice cold PBS. The lysate was prepared by incubating the cells with 1 mL of a lysis buffer (20 mM Tris-HCl, 500 mM NaCl, 1 mM EDTA, 0.1% Tween 20, pH 7.0, protease inhibitor cocktail (Sigma-Aldrich, Saint Louis, MO, USA)) for 30 min on ice followed by sonication, and then, 2 mL of PBS was added. Fluorescence measurements were performed at room temperature in quartz cuvettes with a minimum test volume of 3 mL. The fluorescence intensity of the resulting mixture samples was measured using a K2TM fluorescence spectrometer (ISS, Inc., Champaign, IL, USA) with an excitation filter of 450 nm and emission filter of 510 nm. A minimum of three trials were conducted as previously described [21].

### 2.5. Immuno-Fluorescence Assay

Bm5 cells were cultured on sterile cover slips (placed in 6-well plates) and infected with rBm-His6-SspDnaB-EGFP, rBm-His6-SspDnaB-PPV-VP2, and rBm-His6-SspDnaB-CSFV-E2 at 5 MOI. After 3 days of infection, cells were fixed with cold methanol for 3 min at −20 °C, rinsed with PBS, and blocked with 2% bovine serum albumin for 30 min at 37 °C. Then, the cells were washed three times with PBS. Subsequently, the cells were incubated with specific antibodies against E2 or VP2 for 1 h. After washing the cells with PBS, they were incubated with an Alexa Fluor^®^ 488-conjugated goat antibody (Abcam, Cambridge, UK). Nuclei were stained with PBS containing 10 μg/mL DAPI (Sigma-Aldrich, Saint Louis, MO, USA), and the cells were washed thoroughly. Cover slips were mounted on glass slides with one drop of 50% glycerol in PBS and air dried for 15 min. Visualization and localization of the nucleus and recombinant protein were conducted using a confocal laser scanning microscope LSM 510 (Zeiss, Jena, Germany).

### 2.6. SDS-PAGE and Western Blot Analysis

The cell lysate was prepared by incubating cells with PBST (0.1% Triton-X 100 with PBS) containing a protease inhibitor cocktail (Sigma-Aldrich, Saint Louis, MO, USA) for 30 min on ice followed by sonication, and then, the lysate was mixed with a protein sample buffer and boiled. The protein samples were subjected to 12% SDS-PAGE and transferred to an NC membrane. The membranes were blocked in 5% milk in Tris-buffered saline containing 0.05% Tween 20 and were probed with each of the following antibodies: a GFP monoclonal antibody (Abm, Richmond, BC, Canada), PPV VP2 polyclonal antibody (Biorbyt, Cambridge, UK), CSFV-E2 monoclonal antibody (JENO Biotech, Chuncheon, GW, Republic of Korea), and His Tag monoclonal antibody (Abcam, Cambridge, UK). The membranes were then incubated with a horseradish peroxidase-coupled anti-mouse or rabbit IgG antibody (Cell signaling, Danvers, MA, USA), and the bound antibodies were detected using the enhanced chemiluminescence system (Merck Millipore, Burlington, MA, USA) according to the manufacturer’s instructions.

### 2.7. Temperature and pH Inducible Cleavage

Bm5 cells infected with recombinant virus at 5 MOI were lysed on ice using Buffer B2 (20 mM Tris-HCl, pH 7.0 containing 500 mM NaCl, 0.1% Tween 20 and 1 mM EDTA). The cell lysate was treated at 25 °C for C-terminal cleavage of the Ssp DnaB mini-intein. The incubated samples were loaded on SDS-PAGE gels to confirm the cleavage activity of the Ssp DnaB mini-intein.

## 3. Results and Discussion

### 3.1. Effect of Mini-Intein Fusion Expression

**Figure 2:**
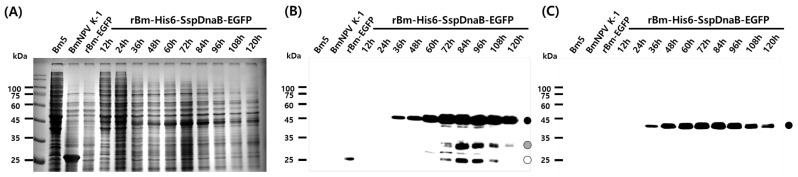
Time course analysis of recombinant enhanced green fluorescent protein (EGFP) fused with the Ssp DnaB mini-intein. *Bombyx mori* 5 (Bm5) cells were infected with 5 multiplicities of infection (MOI) of each virus and harvested from 12 to 120 hours post-infection (h.p.i) at intervals of 12 h. (**A**) Protein samples were analyzed by 12% SDS-PAGE (**B**) and Western blot analysis with an GFP monoclonal antibody (**C**) and His tag monoclonal antibody. Control samples by wild-type BmNPV-K1 and rBm-EGFP were prepared at 72 h.p.i. The black circle represents fusion-expressed EGFP with His6 and the Ssp DnaB mini-intein. The opened circle represents degraded fusion proteins. The gray circle represents EGFP.

The Ssp DnaB mini-intein is a powerful tool in protein engineering research because it can be self-cleaved by controlling the pH and temperature without the use of proteolytic enzymes. Effective purification of a target protein using the self-cleaving activity of the Ssp DnaB mini-intein has been extended to a wide range of applications [22,23,24], for example, purification of Ssp DnaB mini-intein fusion proteins via *E. coli* surface display [25] and purification using elastin-like polypeptide tags [26]. On the other hand, many types of useful proteins have been produced using the BES because it has several advantages over other expression systems. However, there have been no reports on the purification of BES-produced proteins using the SspDnaB mini-intein, probably because of the long time required for the mass production of target proteins in the BES. In general, intracellular self-cleavage is not a problem in the *E. coli* expression system because a sufficient amount of useful protein can be produced over a short time, even if expression is performed at pH 7.0 and 37 °C. Therefore, application of the Ssp DnaB mini-intein in the *E. coli* expression system was not difficult. However, the temperature and pH conditions required by insect cells are nearly identical to the conditions for self-cleavage of the Ssp DnaB mini-intein, and mass production of useful proteins using the BES generally requires more than 72 h. Therefore, in this study, use of the Ssp DnaB mini-intein in the BES was investigated with three different model proteins. Production of EGFP fused with the mini-intein (approximate 45 kDa) was observed at 36 h.p.i and increased with time (Figure 2). Intracellular cleavage of the fusion protein occurred as expected at 72 h.p.i and was visualized by the detection of cleaved EGFP (Figure 2B). The complete fusion protein was maintained for at least 60 h.p.i (Figure 2C). Production of the mini-intein fusion protein was maximal at 72–84 h.p.i.

**Figure 3:**
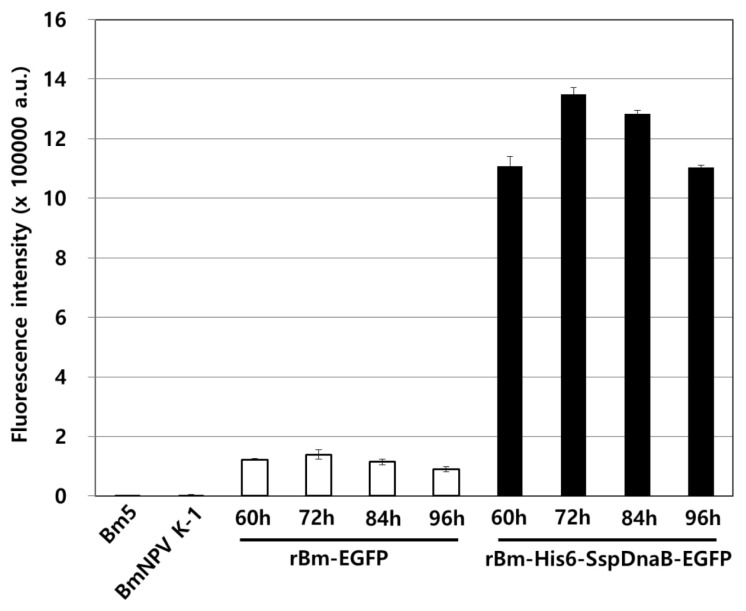
Fluorescence intensity of recombinant EGFP. Bm5 cells were infected with 5 MOI of each virus and harvested from 60 to 96 h.p.i at intervals of 12 h. The fluorescence intensity of the cell extracts was measured using a fluorescence spectrometer with an excitation filter of 450 nm and emission filter of 510 nm. The bars indicate the mean ± SE (*n* = 3).

In addition, fusion with the mini-intein significantly enhanced the production of recombinant EGFP. This effect was clearly shown through a comparison of the fluorescence intensity (Figure 3). The fluorescence intensity of the mini-intein fusion protein was increased by approximately 10 times. This result indicated that the Ssp DnaB mini-intein is sufficient for the purification of recombinant protein in the BES. Despite the production of fusion proteins under similar conditions as mini-intein cleavage, fusion expression of the mini-intein was useful in the BES because of the significantly enhanced production of recombinant proteins by the fusion with the mini-intein. The enhanced production of the fusion protein was enough to overcome the decreased production of the fusion protein due to intracellular self-cleavage. The increase in the expression level of foreign proteins through fusion with the Ssp DnaB mini-intein has been mentioned in the *E. coli* expression system [27], but not in the BES. To examine the optimal culture conditions for the production of the mini-intein fusion protein, the temperature and pH conditions for the culture of insect cells were investigated. To prevent intracellular self-cleavage of the fusion protein, recombinant virus-infected cells were cultured at various pH levels (from 5.8 to 7.0) and temperatures (from 24 to 30 °C). However, changing the cell culture conditions led to worse cell growth and eventually significantly decreased the production of the fusion protein (Figure S1). Therefore, the normal conditions for insect cell culture were more suitable as the optimal conditions for the production of mini-intein fusion proteins.

**Figure 4:**
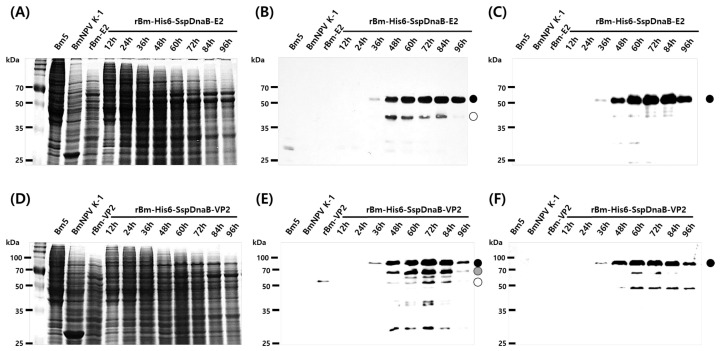
Bm5 cells were infected with 5 MOI of each virus and harvested from 12 to 96 h.p.i at intervals of 12 h. (**A**,**D**) Protein samples were analyzed by 12% SDS-PAGE (**B**) and Western blot analysis with a CSFV-E2 monoclonal antibody, (**E**) PPV-VP2 polyclonal antibody, (**C**,**F**) and His tag monoclonal antibody. Control samples by BmNPV-K1, rBm-E2 and rBm-VP2 were prepared at 72 h.p.i. The black circle represents the fusion-expressed recombinant protein with His6 and the Ssp DnaB mini-intein. The gray circle represents each recombinant protein released from the fusion protein by cleavage of the mini-intein. The opened circle represents degraded target proteins.

The effects of the mini-intein on expression were further examined with two other proteins. The E2 and VP2 proteins are the main antigens of CSFV and PPV, respectively, and were expressed as fusions with the mini-intein. As with EGFP fusion, the production of both fusion proteins was observed from 36 h.p.i and maximum production occurred at a similar time, 72 h.p.i (Figure 4). Fusion with the mini-intein also greatly increased the production of both proteins. However, intracellular self-cleavage of both fusion proteins was observed much earlier, 48 h.p.i (Figure 4B,E). This result indicated that the initiation time of intracellular self-cleavage varied depending on the target protein (Figure 2 and Figure 4). Interestingly, expression of E2 non-fused with the mini-intein was not observed, but expression of E2 was significantly increased when fused with the mini-intein. Unsuccessful expression of recombinant E2 has been reported in similar studies [21,28].

### 3.2. Cleavage Activity of the Mini-Intein

**Figure 5:**
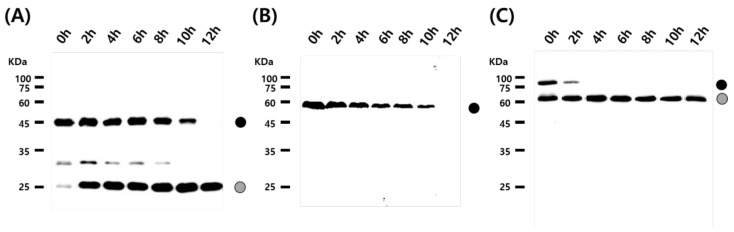
Self-cleavage activity of the Ssp DnaB mini-intein in fusion proteins by temperature and pH adjustments. Recombinant proteins fused with the Ssp DnaB mini-intein were prepared at 72 h.p.i and then treated at pH 7.0 and 25 °C for 12 h. Protein samples were collected at intervals of 2 h and analyzed by (**A**) Western blot with anti-GFP mAb, (**B**) anti-CSFV-E2 mAb (**C**) anti-PPV-VP2 polyclonal antibodies. The black circle represents the fusion-expressed recombinant protein with His6 and the Ssp DnaB mini-intein. The gray circle represents each recombinant protein released from the fusion protein by cleavage of the mini-intein.

To examine the cleavage activity of the mini-intein, the recombinant virus-infected cell lysate was collected at 72 h.p.i and treated at 25 °C and pH 7.0. Cleavage of the EGFP fusion protein began at 2 h and was completed after 12 h (Figure 5A). Cleavage of the VP2 fusion protein occurred at 2 h and was completed after 4 h (Figure 5C). However, production of the E2 fusion protein was reduced but cleaved E2 protein was not observed (Figure 5B). These results suggested the mini-intein fused proteins were successfully cleaved under the proper temperature and pH conditions. Generally, pH 7.0 and 27 °C are suggested as the conditions for the self-cleavage of the Ssp DnaB mini-intein [13,29]. These conditions were also suitable for cleavage of the Ssp DnaB mini-intein fusion protein produced in the BES. The initiation and completion times of self-cleavage differed depending on the fused target proteins (Figure 5). In our study, however, cleavage of E2 was observed as the intensity of the band corresponding to the fusion protein decreases with time but that the cleaved protein was extremely instable and was not detected (Figure 5B). Poor production of E2 has been described in other reports [21].

### 3.3. Status of the Mini-Intein Fusion Protein

**Figure 6:**
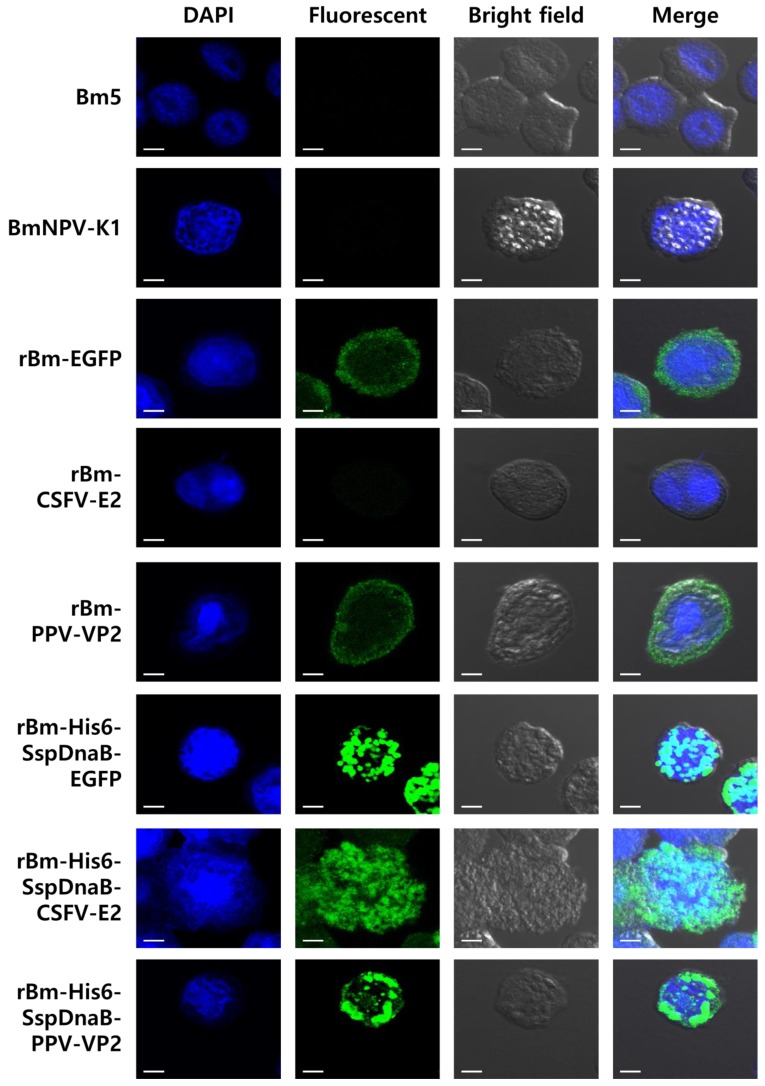
Intracellular distribution of the Ssp DnaB mini-intein-fusion proteins in Bm5 cells. Immunofluorescent staining of the Ssp DnaB mini-intein-fusion and non-fusion proteins was performed using antibodies against E2 or VP2 (secondary goat anti-mouse or rabbit Alexa-Fluor 488, green). Fluorescence was examined in Bm5 cells infected with viruses at three days post-infection. DAPI (2-(4-Amidinophenyl)-6-indolecarbamidine dihydrochloride) staining showing the location of the nuclei. Combined fluorescence and DAPI staining showing the intracellular location of the mini-intein fusion proteins (Merge). The location of recombinant proteins was observed using a confocal laser scanning microscope. Scale bar indicates 10 μm.

To investigate the reason for the enhanced production of the fusion protein, the intracellular status of the fusion protein was examined via immunofluorescence staining. Each recombinant protein except E2 was evenly dispersed in the cytoplasm when expressed in its non-fusion form (Figure 6). However, the mini-intein fused recombinant protein was observed to aggregate in the cytoplasm (Figure 6). The intensity of fluorescence of fusion proteins was also higher than that of non-fusion proteins. Aggregation might increase the stability of the protein against various proteases and may have resulted in the enhanced production of protein. Enhanced stability and production of foreign proteins by supramolecular assembly were reported in other studies [30,31,32,33]. Fusion expression of baculovirus polyhedrin [21,32] and human ferritin heavy chain [33] increased the stability and production of foreign proteins. In our study, the aggregation of foreign proteins did not affect the cleavage activity of the Ssp DnaB mini-intein and His tag affinity purification (Figure S2). However, further study is required to establish whether the work can be successfully translated to a difficult to express protein because this work has been carried out with a model protein. In conclusion, the Ssp DnaB mini-intein is a useful fusion partner in the baculovirus expression system for the easy purification and accumulation of target proteins.

## Figures and Tables

**Table 1:** Primers used in this study.

Name of Primer	Primer Sequence *
His6-SspDnaB-F	5′-GAATTCGCCACCATGCACCACCACCACCACCACATCTCTGGCGATAG-3′
SspDnaB-R	5′-CCATGGCTCTTCCGTTGTGTAC-3′
EGFP-F	5′-GAATTCCCATGGTGAGCAAGGGCGAGGAGCTG-3′
EGFP-R	5′-CTGCAGTTACTTGTACAGCTCGTCCATGCCG-3′
PPV-VP2-F	5′-GAATTCCCATGG**CA**ATGAGTGAAAATGTGGAACAACA-3′
PPV-VP2-R	5′-CTGCAGCTAGTATAATTTTCTTGGTATAAGTTGTG-3′
CSFV-E2-F	5′-GAACCTCCATGG**CA**ATGCGGCTAGCCTGCA-3′
CSFV-E2-R	5′-CTGCAGTCAGTCAGTCACGTCCAGG-3′

* The restriction site incorporated into each oligomer is underlined. The forward PCR primer designed for cloning a given target protein into the system must include the *Nco* I site and the subsequent CA nucleotide sequence before the annealing portion on the target, which allows for the construction of a proper in-frame fusion.

## Supplementary Materials

The following are available online at http://www.mdpi.com/1999-4915/10/10/523/s1.
