# Supplementary material for: Enhanced Production of Recombinant Protein by Fusion Expression with Ssp DnaB Mini-Intein in the Baculovirus Expression System
Source: Viruses. 2018 Sep 25;10(10):523. doi: 10.3390/v10100523 (PMC6213604; doi:10.3390/v10100523)
Supplement: Supplementary file 1 [file viruses-10-00523-s001.pdf]

Supplementary Data

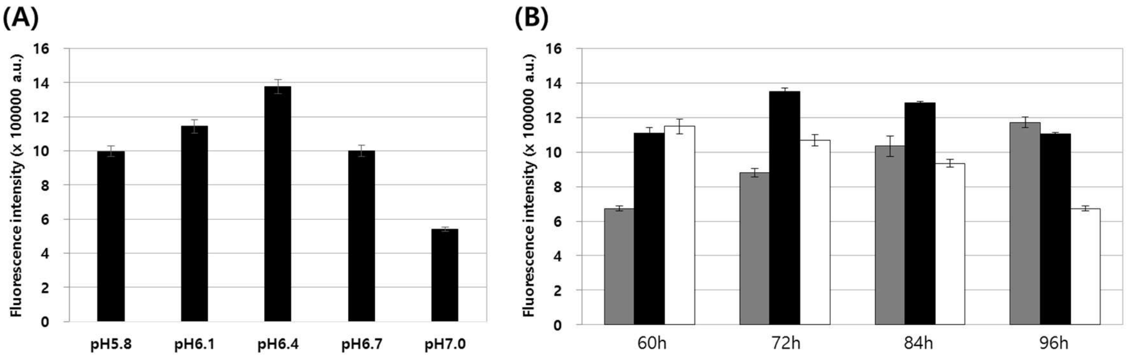

Figure S1. Fluorescence intensity of recombinant EGFP according to the pH and temperature in cell culture conditions. Bm5 cells were maintained at various pH (A) and temperature (B) conditions and were infected with 5 MOI of rBm-His-SspDnaB-EGFP. Virus-infected cells were harvested and the fluorescence intensity of the cell extracts was measured using a fluorescence spectrometer with an excitation filter of 450 nm and emission filter of 510 nm. Under the various pH conditions, the temperature was adjusted to 27 °C and cells were harvested at 72 h.p.i. The pH was adjusted to 6.4 when the culture temperatures were changed to 24 °C (gray), 27 °C (black) and 30 °C (white). The bars indicate the mean ± SE (n=3).

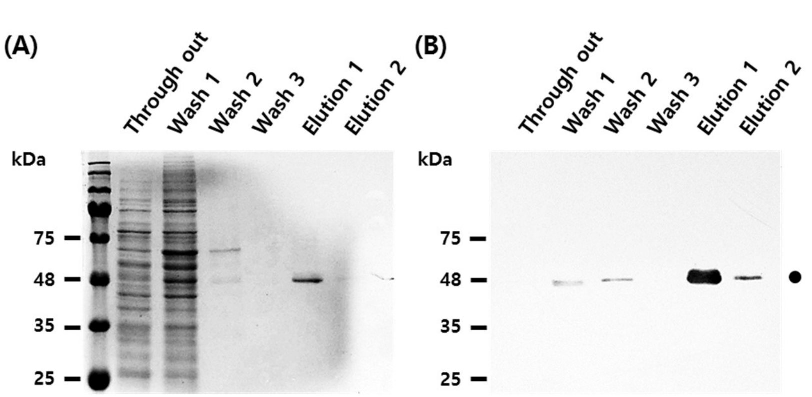

Figure S2. Purification of His6-SspDnaB-EGFP using His tag affinity column. Bm5 cells infected with rBm- His6-SspDnaB-EGFP at 5 MOI, 60 h.p.i were lysed on ice using RIPA Buffer (without EDTA). Total cell lysate was passed through a His tag affinity column (elpisbio, Deajeon, Republic of Korea) according to the manufacturer's manual and the fractions were collected. The collected fractions were loaded on a 12% SDS-PAGE gel (A). Western blot analysis against EGFP was then performed using GFP monoclonal antibody (B). The black circle represents the fusion-expressed EGFP with His6 and the Ssp DnaB mini-intein.
